# Editorial

**Published:** 1863

**Authors:** 


### Editorial.

### ILLINOIS STATE MEDICAL SOCIETY.

The Eleventh Annual Meeting of the Illinois State Medical Society convened in the Old Presbyterian Church, in Jacksonville, at ten o'clock A.M., of May 5th, 1863.

The meeting was called to order by Dr. D. Prince, Chairman of the Committee of Arrangements, and in the absence of the President and permanent Secretary, Dr. H. Noble, of Heyworth, was appointed temporary Chairman, and Dr. M. Shepherd, temporary Secretary.

The Committee of Arrangements reported the following delegates and new members present, viz.:

Dr. A. McFarland, of Jacksonville, Illinois,
" J. M. Steele, of Grandview, Illinois,
" F. B. Haller, of Vandalia, "
" M. Shepherd, of Payson, "
" A. H. Luce, of Bloomington, "
" H. Noble, of Heyworth, "
" N. English, of Jacksonville, "
" H. Jones, of " "
" D. Prince, of " "
" J. Frazer, of Howard's Point, Illinois,
" N. Wright, of Chatham, "
" W. M. Landon, of Burton,
" R. E. McVey, of Waverly,

" B. Wilson, of Chambersburg, Illinois,
" Josh. Rhoads, of Jacksonville, "
" A. P. Tenney, of " "

Dr. Prince, in behalf of the Committee of Arrangements, submitted the following order of business:

First day, morning session devoted to business. Afternoon session, from 2 to 4 o'clock, devoted to business. At 4 o'clock P.M., visit the Institution for the Blind.

Second day, morning session devoted to business. 2 o'clock P.M., dine at the Hospital for the Insane. 4 o'clock P.M., visit the Asylum for the Deaf and Dumb.

Dr. A. H. Luce moved the appointment of a committee of one from each county represented, to nominate officers for the ensuing year.

Pending this motion the following additional members arrived and took their seats, viz.:

Dr. N. S. Davis, of Chicago, Illinois,
" E. L. Holmes, " "
" J. H. Hollister, " "
" E. Andrews, " "
" J. R. Askew, of Jacksonville, Illinois.

The motion of Dr. Luce was then adopted, and the following Committee on Nominations appointed, viz.:

Dr. F. B. Haller, of Fayette county, Illinois,
" A. H. Luce, of McLean " "
" E. Andrews, of Cook " "
" M. Shepherd, of Adams " "
" N. Wright, of Sangamon " "
" A. McFarland, of Morgan " "
" B. Wilson, of Pike " "
" J. M. Steele, of Edgar " "

The Minutes of the last Annual Meeting were read by the permanent Secretary, N. S. Davis, and approved.

An invitation to visit the Illinois Institution for the Blind, at 4 o'clock P.M., was received from Dr. Joshua Rhoads, Superintendant, and accepted by a vote of the Society.

Reports from Standing Committees were called for, and the committees called in order as follows:

Committee on Practical Medicine. No report.
do on Drugs and Medicines. No report.
do on Obstetrics. No report.

Committee on Surgery being called, in the absence of the Chairman, Dr. A. W. Heise, Dr. E. Andrews stated that he had partially prepared a report, an abstract of which he would present during the afternoon session.

Dr. D. W. Stormont, formerly of Grandview, Chairman of the Committee on Registration of Births, Marriages and Deaths, sent the following report, which was read by the Secretary, viz:

The Committee on the Registration Law would report, that early in January the petition of this Society and a bill for a law, were forwarded to Hon. T. A. Marshall. They were presented to the Senate, and referred to the Committee on Judiciary, where they still remain in spite of all our entreaties.

In the present distracted condition of the country, we cannot recommend any measures as likely to be efficient in securing the passage of this law. Hygeia is deposed; Mars now reigns supreme. Perhaps at our next annual meeting this condition of things will be reversed.

Respectfully submitted, D. W. STORMONT,
*Chairman Com.*

On motion of Dr. F. B. Haller, the report was accepted and the committee discharged.

Dr. D. Prince, Chairman of the Committee to petition the Legislature in favor of provisions for the training of idiotic and imbecile children, reported, that in consequence of the condition of the country, nothing had been done by the committee during the past year. On motion of Dr. Hollister, the committee was continued.

Dr. E. L. Holmes, in behalf of the Committee on Prize Essays, reported, that the only paper in the hands of the Committee was the one referred to at the last meeting; and on it he was not authorized to report by the other members of the Committee. On motion the Committee was requested to complete the examination of said paper, and report the result to the Committee of Publication, as early as possible.

The annual report of the Treasurer, Dr. J. W. Freer, was presented by Dr. Holmes, accepted and referred to an auditing committee, consisting of Drs. E. Andrews, E. L. Holmes, and F. B. Haller.

Dr. J. D. Grissim, of Jacksonville, was proposed for permanent membership, by Dr. N. English, and unanimously elected.

On motion the Society adjourned to 2 o'clock P.M.

AFTERNOON SESSION.

Dr. Noble, temporary Chairman, called the Society to order at 2 o'clock P.M.

The Committee on Nominations made the following report, which was accepted, and the officers named unanimously elected:

OFFICERS.

*For President.*—Dr. A. McFarland, of Jacksonville.

*For Vice-Presidents.*—Dr. A. H. Luce, of Bloomington; Dr. A. Frazer, of Howard's Point.

*For Treasurer.*—Dr. J. H. Hollister, of Chicago.

*Committee on Practical Medicine.*—Dr. N. S. Davis, of Chicago; Dr. T. D. Washburn, of Hillsboro; Dr. C. R. Park, of Bloomington.

*Committee on Drugs and Medicines.*—Dr. F. K. Bailey, of Joliet; R. G. Laughlin, of Hayworth; F. R. Paine, of Marshall.

*Committee on Obstetrics.*—Dr. DeLaskie Miller, Chicago; Dr. J. M. Steele, Grandview; Dr. A. H. Luce, Bloomington.

*Committee on Surgery.*—Dr. E. Andrews, Chicago; Dr. S. W. Noble, LeRoy; Dr. F. B. Haller, Vandalia.

The Chairman appointed Drs. Steele, Luce, and Hollister, a committee to conduct the officers elect to their places.

Dr. E. Andrews, from the Committee on Surgery, read an abstract of his report, which was accepted and the report referred to the Committee of Publication.

Dr. D. Prince read a very interesting paper on "Delayed

Union of Fractures of the Bones." Also, remarks on amputations, etc., comparing the advantages of circular and flap operations, especially in military surgery. Remarks in reference to certain points in the paper, were made by Drs. E. Andrews, and J. H. Hollister.

Adjourned to the Blind Asylum, and to evening session at 7½ o'clock.

EVENING SESSION.

Society was called to order by the temporary Chairman, at 7½ o'clock P.M. Dr. A. McFarland, the President elect of the Society, was conducted to the chair by the committee. On taking his official position, Dr. McFarland accepted the office in a brief but very appropriate address, welcoming the members cordially to the hospitalities of the city of Jacksonville.

The paper read by Dr. D. Prince being under consideration, it was further discussed by Dr. Andrews, particularly in reference to the comparative value of resections and amputations of the shoulder and elbow, showing by statistics, that resections of these joints was attended by less mortality than the amputations. Resections of the femur uniformly fatal. Resections of the knee-joint yet doubtful in its results. Dr. Prince continued the discussion in explanation of the causes which render resections of the shoulder and elbow more safe than amputation. It was because such resections caused less wounding of the soft areolar tissues than amputations would. But in most cases of wounds of the knee-joint, the resections must cause more injury of that class of tissues than amputation of the thigh a little above.

On motion of Dr. Hollister, the paper of Dr. Prince was accepted and referred to the Committee of Publication.

The Society was invited to hold its next meeting at Chicago and also at Quincy.

Dr. E. L. Holmes read an interesting report on Diseases of the Eye, relating particularly to the cases of catarrhal ophthalmia. Dry air, severe winds, dust in the atmosphere, and contagion were mentioned as the causes of chief importance. Sympathetic ophthalmia, or that which is induced in one eye by severe injury previously to the other eye, is of the most dangerous and persistent character. Recommended early extirpation of the injured eye as a preventive. Also related three cases in which chloroform produced unpleasant effects, but no deaths. Glaucoma was commented on somewhat in detail.

On motion of Dr. H. Noble, the report was accepted, and, after a brief discussion by Dr. B. Wilson, Dr. J. M. Steele, Dr. Prince, Dr. Haller, and Dr. Frazer, it was referred to the Committee of Publication.

Dr. N. S. Davis, in behalf of the Publishing Committee, submitted the following report, viz.:

REPORT OF THE COMMITTEE OF PUBLICATION, 1861.

As soon as the papers read at the meeting of the Society in May, 1860, could be collected, the *Transactions* were put to press. Owing to the unusual length of the report on Surgery, it was evident that if the whole expense of publication was incurred by the Committee, it would much exceed the amount of funds in the hands of the Treasurer of the Society. To avoid this result as far as possible, the undersigned caused a large part of the matter contained in the *Transactions* to be first published in the *Chicago Medical Examiner*, by which, the expense of setting the type was saved to the Society. Notwithstanding every effort, however, to reduce the cost of publication of the *Transactions*, the sum exceeded the amount in the treasury, as shown in the Treasurer's Report.

The number of copies published was 300, containing 226 pages each, at a cost of $192.00. Sixty copies have been distributed to such members as were entitled to the same by the payment of their annual assessment, twenty copies have been sent to medical journals and other State medical societies, and thirty copies were brought here for the use of members at this meeting, leaving in possession of the Society about 175 copies for further use. At the meeting in 1860, the Secretary was requested to have a revised copy of the Constitution and By-Laws re-published with the *Transactions;* but owing to the insufficiency of the funds they were omitted. As the previously published copies are nearly exhausted, it is suggested that they be published with the *Transactions* of the present meeting. The present indebtedness of the Society for printing, as shown by the Treasurer's Report, is $70.50. The Secretary has advanced $4.69 in prepaying postage on copies of *Transactions* already distributed, and which he freely donates to the Society. In notifying the present meeting, care was also taken to send notices to each individual member whose name was in the preceeding volume of *Transactions*, and to all members of committees. All of which is respectfully submitted in behalf of the Publishing Committee by

N. S. DAVIS, *Permanent Sec'y.*

*May 5th*, 1863.

On motion of Dr. J. M. Steele, the report was accepted and approved, and the thanks of the Society tendered to the Secretary for the judicious manner in which he had discharged the duties of the Publishing Committee.

Drs. C. A. Fisher and C. H. Knight, of Jacksonville, were proposed for permanent membership, by Dr. D. Prince, and unanimously elected. Dr. M. T. DeWitt, of Whitehall, was also proposed by Dr. D. Prince, and unanimously elected. Dr. G. W. Albin, of Neoga, was proposed by F. B. Haller, and elected unanimously.

On motion the Society adjourned until 8 o'clock in the morning.

SECOND DAY.—MORNING SESSION.

The President in the chair called the meeting to order at 8½ o'clock A.M. Secretary read the minutes of yesterday, which were accepted and approved. Committee on nominations made the following report, which was accepted and adopted:—

The Committee on Nominations report the assessment for this year to be two dollars for each member. For place of next meeting, Chicago; for time, the first Tuesday in May. For Committee of Arrangements, Drs. M. O. Heydock, Thomas Bevan, E. L. Holmes, Chas. G. Smith, S. Wickersham, J. W. Freer, J. H. Hollister. For Assistant Sec., H. W. Jones.

The following additional gentlemen were proposed as permanent members, by Dr. D. Prince, and unanimously elected, viz.: C. M. Robertson, of Tallula, Menard county, and Hiram R. Jones, of Jacksonville.

Dr. D. Prince was appointed a special committee to report, at the next annual meeting, on Orthopœdic Surgery.

Dr. N. S. Davis offered the following resolutions, which were unanimously adopted:—

*Resolved*, That in compliance with the invitation of the Faculty of the Chicago Medical College, and Medical Department of the Lind University, the President of this Society shall appoint a board of three Censors, to attend the examination of candidates for graduation at the public annual commencements in that institution, and to participate in such examinations by questions and votes.

*Resolved*, That the Society publish, in the preface and title page of the next volume of *Transactions*, an explanation of the omission of two years in the meetings and doings of the Society.

Dr. H. Noble read an interesting paper on the Nature and Treatment of Typhoid Fever, which was accepted and discussed by Dr. D. Prince, who stated that in the Army of Virginia, quinine was never found beneficial, and was sometimes injurious in true typhoid fever.

The reading of Dr. Noble's report was followed by a very interesting discussion on the treatment of typhoid fever, which was participated in by Drs. Prince, Davis, Steele, Andrews, and Noble, at the close of which, the report was referred to the Committee of Publication.

On motion of Dr. Davis, the Society proceeded to the election of fifteen delegates, to represent it in the approaching meeting of the American Medical Association. The following named gentlemen were duly elected, viz.:—

M. Shepherd, of Payson; J. M. Steele, of Grandview; F. B. Haller, of Vandalia; David Prince, of Jacksonville; H. Noble, of Heyworth; Lucius Clark, of Rockford; A. H. Luce, of Bloomington; John Tenbrook, of Paris; M. F. Dewitt of

Whitehall; F. R. Payne, of Marshall; L. L. Todd, of Paris; N. Wright, of Chatham; S. W. Noble, of LeRoy; Samuel Thompson, of Albion; A. J. Crane, of Decatur.

Dr. Hollister offered the following resolution which was unanimously adopted:—

*Resolved*, that we tender to those of our members serving as medical officers in the Army, and now absent in the field, assurances of our kind remembrance of their humane labors and personal sacrifices; that we congratulate them upon their being able to contribute services so valuable to a cause so *noble*, and that we join with them in earnest desires, and personal efforts if need be, for the early restoration of *peace* and the integrity of the *entire* Union.

Dr. M. Shepherd proposed the following resolution, which was adopted:—

*Resolved*, that Prof. Davis, if not incompatible with his other duties, be requested by this meeting to write an essay upon the Deleterious Effects of the use of Tobacco upon the Human System, and especially upon the example and bad taste of the members of our profession who habitually use it.

At half-past twelve o'clock, the Society adjourned to the Hospital for the Insane, where they were cordially received by the superintendent, Dr. A. McFarland, and his assistant, Dr. Tenny. The superintendent briefly explained the rules for the admission of patients, the system of management adopted in in every department, the number of patients at present in the institution, and the whole number received since it was established. The company then sat down to an excellent dinner, after which, they were conducted through all the wards for patients, and all the departments of husbandry connected with the outdoor management of the extensive grounds connected with the hospital. Everything, within and without, was found to exhibit the most perfect order and the highest degree of good taste and skill on the part of the superintendent. After completing the examination of the premises, the party assembled in the reception-room, and were called to order by Dr. A. H. Luce, Vice-President. Dr. McFarland read a very interesting paper on "Minor Mental Maladies," which was received and referred to the Committee of Publication, and a vote of thanks tendered to the author.

At four o'clock P.M., the members of the Society again took the omnibuses and proceeded to the Institution for the education of the deaf and dumb. Here, as in other public institutions, they were cordially welcomed and conducted through the building, the work-shops, and all places of interest, after which, they assembled in the chapel-room and witnessed some intensely interesting exercises, showing the progress of the pupils and the modes of their instruction. The Society then adjourned to meet at 7½ o'clock P.M., in the Dunlop House.

EVENING SESSION.

Dr. A. H. Luce, Vice-President, called the Society to order at 7½ o'clock P.M.,

Dr. James Leighton, of Manchester, Ill., was proposed for permanent member by Dr. Prince, and unanimously elected.

The following resolutions were read by the Secretary and adopted by unanimous vote:—

*Resolved*, that the members of this Society have been highly pleased with their visit to the Illinois Institution for the education of the blind. The proficiency of the pupils in their studies, their excellent sanitary condition, and the neatness and order exhibited in every department, from the kitchen to the dormitories, reflect the highest credit on the superintendent, matron, and teachers.

*Resolved*, that the thanks of the Society are hereby tendered to the superintendent and matron, Mr. and Mrs. Rhodes, for the kind hospitalities extended to its members.

*Resolved*, that the thanks of the Society are hereby tendered to the Committee of Arrangements, for the excellent accommodations and arrangements for our enjoyment and profit at the present meeting.

*Resolved*, that our visit to the Hospital for the Insane has been exceedingly pleasant and satisfactory in all respects, and that we tender to the accomplished superintendent and his assistant our thanks for their kind hospitality.

*Resolved*, that the examination of the institution for the education of the deaf and dumb, and the exercises of the pupils, has been most gratifying to all of us; and we cordially thank the officers and teachers for their kindness, and commend them in the prosecution of their arduous and noble work.

*Resolved*, that the thanks of the Illinois State Medical Society be extended to the 2d Presbyterian Church of Jacksonville, for the use of its church edifice; and that the Secretary be instructed to furnish a copy of this resolution to its Board of Trustees.

On motion, Dr. A. McFarland was appointed a delegate to represent this Society in the next annual meeting of the New Hampshire State Medical Society.

Dr. N. S. Davis made a verbal communication to the Society on the condition of the blood in malignant typhus, scarlatina, variola, and erysipelas, and related several cases of the latter disease, in the treatment of which the *sulphite of lime* was given in doses of one drachm every two or three hours with apparent benefit. After a brief discussion, Dr. Davis was requested to furnish the substance of his remarks for publication in the *Transactions* of the Society.

At 9 o'clock P.M., the Society adjourned until the first Tuesday in May, 1864.

N. S. DAVIS, *Secretary*.

---

ILLINOIS STATE MEDICAL SOCIETY.—We publish, in the present number, a full record of the proceedings of the recent meeting of the State Medical Society, at Jacksonville. It was one of the most pleasant and profitable meetings that have been held since the organization of the Society. The communications made to the Society were not numerous but well prepared, and of much practical value. The topics embraced were discussed with more freedom and interest than usual. The visits to the several public institutions were gratifying to every member present. Much credit is due to the Committee of Arrangements for the judicious management of the meeting.

EXPLANATION.—The reader will readily perceive that the present number of the *Examiner* is increased in size, and thereby made to include both the months of March and April. We shall issue another double number in June, and thereby bring the future monthly issues to their proper date, where we shall endeavor to keep them hereafter. We have several valuable papers for the original department of the number for May and June. It will also contain a full account of the proceedings of the American Medical Association during its coming session in this city.

---

CHARACTERISTIC.—The editors of the *Chicago Medical Journal*, Drs. Brainard and Allen, are still doing all they can to discourage attendance on the meeting of the American Medical Association, and misrepresent the action of the Committee of Arrangements. All their efforts, however, will be unavailing. The meeting will be largely attended, well provided for, and equal in interest and importance to any that have preceded it.

---

### CALOMEL AND TARTAR EMETIC IN THE ARMY.

We clip the following singular order, recently issued by the Surgeon-General of the United States Army, from a daily paper; the editor of which, very pertinently asks, whether, instead of excluding two important articles of the *Materia Medica*, it would not have been better to have dismissed those Surgeons and Assistant-Surgeons who did not know enough to use the remedies judiciously?

*Surgeon General's Office,*

WASHINGTON, D. C., *May* 4, 1863.

1. From the reports of medical inspectors, and the sanitary reports to this office, it appears that the administration of calomel has so frequently been pushed to excess by military surgeons as to call for prompt steps by this office to correct this abuse; an abuse the melancholy effects of which, as officially reported, have exhibited themselves not only in innumerable cases of profuse salivation, but in the not infrequent occurrence of mercurial gangrene.

It seeming impossible in other manner to properly restrict the use of this powerful agent, it is directed that it be struck from the supply-table, and that no further requisions for this medicine be approved by medical directors. This is done with the more confidence, as modern pathology has proved the impropriety of the use of mercury in very many of those diseases in which it was formaly unfailingly administered.

2. The records of this office having conclusively proved that diseases prevalent in the army may be treated as efficiently without tartar emetic as therewith, and the fact of its remaining upon the supply-table being a tacit invitation to its use, tartar emetic is also struck from the supply-table of the army.

No doubt can exist that more harm has resulted from the misuse of both these agents in the treatment of disease, than benefit from their proper administration.

W. A. Hammond, Surgeon-General.

---

Cure of Bright's Disease by a Milk Diet.—A writer in the *Bulletin de Therapeutique* for March, over the signature of F., in an article on this subject, contends that the disorganization of the kidneys known by this name is a secondary affection, dependent upon the albuminuria and caused by it. The primitive disease, he contends, is in the blood itself, and consists in a modification of the qualities of the albumen of the serum, at present not understood, by which it passes through the pores of the vessels. Some plausibility would seem to be given to this theory by the known efficacy of astringents, such as the salts of iron, tannin, &c., in many cases. The writer argues that if the condition of the blood can be changed the disease of the kidneys will be cured. To effect this he strongly recommends a milk diet. From the article, which is too long for us to give entire, we translate the following extract:

We know that the use of milk as an exclusive diet is a very old remedy for dropsy, whatever may be its cause. Horstius, Hilden, Bontius, Mauriceau, &c., have successively vaunted the efficacy of this treatment, which, like so many other good things in popular use, was neglected until 1831, when M. Chrestien, of Montpellier, brought this remedy to honored notice, and proved that it would be often successful when all other means failed.—M. Serres, d'Alais, Claudot, Ossieur, Dieudonne, &c., have since arrived at conclusions entirely confirmatory of those reported by Chrestien, and the *Bulletin de Therapeutique* has not failed to report with all fidelity all the facts which have been brought forward in favor of this remedy. The articles referred to regarded ascites or anasarca only in a general manner, and as a condition, independently of the cause which produced it; and although we must admit that in these results the dropsy of albuminuria may claim a certain number of cases, it had never been studied in a special manner with reference to a milk diet, up to the time of the very remarkable article of M. Guignier, Member of the Faculty of Montpellier, published in the *Bulletin de Therapeutique*, vol. liii., p. 337, and that published by M. Artigues in the *Jour. de Med. et Chirurgie Militaria.* M. Guignier applied himself with commendable assiduity to the specification of the cases in which the method of Chrestian was indicated or the reverse. He believed it useful when there exists a condition of plethora, hurtful, on the contrary, when the dropsies are of a passive character, as the patients are too debilitated for it. It would be difficult for us to judge of the value of these distinctions and the possibility of defining them in the greater number of cases; and we can very readily believe that if the dropsies of plethora are best adapted to a milk diet, it is only because they are attacked at an earlier stage, and that the constitution of the patient offers then more resources. M. Guignier furthermore does not give the first place to the method of Chrestien, but to that of M. Serres, d'Alais, based as we know on the combination of these three methods: 1st, the diminution of the amount of fluid drank; 2d, a milk diet; 3d, the use of raw onions. He lays down his treatment as follows: to chose the milk with special care as regards its freshness and quality, to change it for a supply from another source when the first fails to agree with the patient; to allow it to be taken *ad libitum* (this practice differs from that of M. Serres); to combine chalk or magnesia with it when it excites acidity; to give up this treatment at the end of twenty days when it fails to produce a marked improvement. M. Guignier, without attaching much importance to the use of raw onions, nevertheless thinks that their diuretic property is useful, and that when the stomach tolerates them they should make a part of this milk regimen.

The facts offered by M. Guignier were of much weight in favor of a milk diet, and while accepting them with some reserve, in view of the possibility when once the dropsy is absorbed of seeing the albuminuria which produced it still remain, yet the impression could not be resisted that milk affords a means of treatment of the greatest value in albuminuria. The observations published by M. Artigues, physician-in-chief of the army, furnish evidence even more decisive in favor of this regimen.—He cites, in fact, two cases of complete recovery, without any return of symptoms for three years. Mr. Serres's method was that followed by M. Artigues without any variation; it is that also which ought, in our opinion, to be followed in the treatment of Bright's disease; it combines, in effect, the advantages of a dry and a milk diet.

Shall we not be able, by this purely dietetic treatment, to cure radically these formidable affections? We do not doubt it, if the treatment is employed in good season, when the effusion is recent and the albuminuria has not existed long enough to have produced in the kidneys the organic lesions which are evidently beyond our resources. But even in this case, where our art is reduced to a deplorable helplessness, the following a strict regimen, of a dry and vegetable diet, but above all a milk diet, will give more favorable results than any medication known as yet. We have, therefore, felt it our duty to call attention to these means, which, besides, are recommended by their perfect harmlessness.—*Boston Medical and Surgical Journal.*

---

Death of Dr. Charles Hooker.—It is with regret that we announce the death of the distinguished physician, Dr. Charles Hooker, of New Haven, on the 19th of March, at the age of 64. The deceased had been a supporter of our *Journal* almost from its beginning, and in past years communicated many valuable articles for our pages. From an obituary in the *New Haven Daily Palladium*, we make the following extract:—

"Dr. Hooker was born in Berlin, in this State, a descendent of that eminent and gifted man who was the leader of the first settlers of Hartford, the Rev. Thomas Hooker. He graduated with honor in Yale College, in 1820, in the class of which President Woolsey and Dr. Bacon were members. On graduating, as he afterwards did from the Medical Institution of the College, he began practice in this city, and from that time to this he has been known as one of the busiest and most indefatigable men in this community. In 1838 he was appointed to the chair of Anatomy and Physiology, and the numerous graduates of the Medical School can testify to his great skill and energy as a teacher.

"The character of Dr. Hooker was not a common one. An independent thinker, his energy prompted him to press his views upon the minds of others, and he therefore made a decided impression upon the principles and practice of his brethren in the profession. No man ever showed more earnestness and assiduity in his calling, and these were just as manifest in his last days, when most men incline to some relaxation of their labors, as they were when the ardor of his youth was upon him. Faithful and energetic to the last, he exposed himself freely to cold and fatigue, in behalf of some patients in whom he felt a deep interest, even after his sickness had fairly begun, and so he may be said to have died in the very midst of his labors. What we deem to be the grand fact of his professional life, standing out prominent before all others, and written in deep lines upon the hearts of multitudes in this community, is, that he performed his labors for the sick irrespective of reward; for he was just as ready to obey the calls of the poor as those of the rich. The genial and ardent social qualities of Dr. Hooker added much to his influence, and therefore his usefulness as a physician. We may add to this brief notice the fact that he was a member, for we know not how many years, of St. Paul's Church, and none will more deeply feel his loss than the pastor and members of that church."—*Boston Medical and Surgical Journal.*

---

Death of Dr. Charles Fishback.—It is with deep and sincere sorrow that we announce the death of Dr. Fishback, of Indianapolis. We had hoped to receive a full obituary notice from some of our Indiana friends, but have been disappointed. His death was from a dissecting wound, received, as we understand, in making a *post mortem* examination of a puerperal fever case, but have no definite particulars.

The death of Dr. Fishback is a loss to the profession of Indiana. He was high-toned in all his opinions and practice, one of the most active and zealous members of the State Society, an ardent worker for the elevation, educationally, of his profession, and, above and beyond all, a sincere Christian gentleman.—*Cincinnati Lancet and Observer.*

---

Obituary Record —Died, at his country-seat, at Fordham, Feb. 14, of pleuro-pneumonia, Geo. P. Cammon, M.D., aged 59 years. Dr. C. held a very high position in the estimation of his brethren in New York, and was considered as among the most advanced and accurate students of pulmonary and cardiac diseases in our country. Though possessed of great wealth, he was most punctual and faithful in his attendance at the New York Dispensaries to which he was attached. Owing to his aversion to publicity, he has published but little, and seldom mingled in professional circles; but he was universally respected, and regarded as a bright ornament of the profession.—*Medical News and Library.*

RESIGNATION OF PROF. H. H. CHILDS.—At a meeting of the Trustees of the Berkshire Medical College, held last Thursday, Hon. Henry H. Childs, the President of the Institution, as well as its founder and father, resigned the Professorship of "Obstetrics and the Diseases of Women and Children," which he has held so many years. Dr. Childs' advanced age rendered it necessary that he should seek some relief—although a hale and hearty old age is his, which we trust will enable him to hold for years the Presidency of the College, which he still retains. In accepting the resignation the Board adopted unanimously the following resolutions:

*Resolved*, That the resignation of Dr. Childs requires from us more than a passing notice. For nearly forty years he has been the active head of the Berkshire Medical College—his usefulness having extended to a period almost unprecedented. During these years, by his energy, zeal, and enthusiasm, he has achieved a wide-spread reputation as a medical man, and by his kindness of heart, and courtesy of manner, a no less deserved name as a Christian gentleman. He has ever maintained a high standard of medical honor, and his pupils must forget or ignore his teachings before they could stoop to anything ignoble or ungenerous. With a quick appreciation of merit, however modest, and ever ready with the kindly word of needed encouragement, his pupils learned to love him, and thousands, through the length and breadth of our land, affectionately look back to him as a kindly foster-father.

While we regret the infirmities which compel the retirement of our venerable President, as an active instructor, we earnestly hope that his interest in the institution, which is so identified with his life and name, may not abate, and that he may long be spared to speak words of cheer to the new generation of students, and to give the benefit of his advice and counsel to the Faculty and Trustees.—*Buffalo Medical and Surgical Journal.*

---

WASHINGTON CITY, the National Capital, is undoubtedly in the most insanitary condition of any city in the United States. The principal sources of uncleanliness are thus given by Dr. Henry G. Clark, of the Sanitary Commission, in a letter to the military authorities, recommending the adoption of appropriate measures:—

"1st. The accumulation of large numbers of men and animals in confined locations. 2d. The accumulations of filth, such as vegetable and animal offal, consequent on the above. 3d. The entire neglect of cleansing operations in the yards, lanes, and streets of the city, especially the very deficient drainage. 4th. The nuisance of a shallow, and neglected, and filthy canal in the heart of the city, a receptacle of the sewers, and a place of deposit for dead horses, etc. 5th. The marshy and stagnant water in many vacant lots, some of them—as in North Capitol street—near large hospitals, the want of drainage of which has rendered many parts of the city, as that near the President's House, malarious spots, producing intermittent and remittent fevers, jaundice, etc. 6th. The accumulation of the sick in large numbers is a very powerful means, unless proper sanitary measures are taken, of intensifying all the ordinary and extraordinary causes of disease."

The recommendations of Dr. Clark embrace a rigid system of sanitary police, which the nation is interested in seeing enforced.—*Medical Times.*

---

Sulphate of Morphia administered through the Ear.—Dr. W. H. Traver, of Providence, R. I., writes as follows:

Mrs. C., a lady of nervous and excitable temperament was awakened from a sound sleep by a severe neuralgic pain in her right ear.

After looking in vain for a vial containing tincture of opium, some of which she had used in her ear on a former occasion, she found a powder of sulphate of morphia, (one-fourth grain) which her husband in compliance with her request put in her ear.

The pain stopped and she soon became much surprised and not a little alarmed to find herself under the influence of the drug, which, however, passed off in due time without any inconvenince.—*Medical and Surgical Reporter.*

---

How they Live in New York.—The New York *Sun* says, that there are in that city 12,347 tenement houses, containing a total population of 401,376 persons—an average of about 33½ to each house. Of this number—a good sized town of itself—22,095 live in cellars, some of them scarcely fit for brutes.—The ventilation in about one-third of these houses is bad, and of course so far injurious to health. In case of fire, &c., 8546 houses, containing a population of 253,901 souls are provided with good means of escape, while 3801 houses, with a population of 125,280, are deficient in this respect.—*Medical and Surgical Reporter.*

